# Acute transplant renal artery thrombosis due to distal renal artery stenosis: A case report and review of the literature

**DOI:** 10.12860/jnp.2014.20

**Published:** 2014-07-01

**Authors:** Mohammad Kazem Fallahzadeh, Rajini Kanth Yatavelli, Ajay Kumar, Neeraj Singh

**Affiliations:** ^1^Division of Nephrology, Department of Medicine, Louisiana State University Health Sciences Center-Shreveport, Shreveport, Louisiana, USA; ^2^John C. McDonald Transplant Center, Willis-Knighton Health System, Shreveport, Louisiana, USA

**Keywords:** Renal artery thrombosis, Doppler ultrasound, Graft loss, Thrombectomy

## Abstract

*Background:* Acute renal artery thrombosis is a devastating complication of renal transplantation that can result in graft loss if not detected early. Surgical and technical errors are the major cause of renal artery thrombosis. In this article, for the first time, we are reporting a case of acute renal artery thrombosis that developed early post-transplantation due to distal renal artery stenosis.

*Case Presentation:* A 71-year-old woman presented with nausea, vomiting and decreased urine output 7 days after a deceased donor kidney transplant. Doppler ultrasound showed absent renal and venous flow in the transplanted kidney. Transplant renal artery angiogram showed renal artery thrombosis. Catheterization and thrombectomy were done in the occluded renal artery. After thrombectomy, renal angiogram showed distal renal artery stenosis which was dilated by stenting. Post-stenting angiogram showed good blood flow in the entire renal arterial system. However, the patient^,^s kidney function did not improve within next 24 hours and the patient eventually lost the kidney. Kidney biopsy showed widespread kidney infarction with no evidence of rejection.

*Conclusions:* Our case shows that renal artery thrombosis can develop due to distal renal artery stenosis and if not detected early could result in graft loss.

## 1. Introduction

Acute renal artery thrombosis is a catastrophic complication of kidney transplantation that can result in graft failure if not detected early (1). Surgical and technical errors are among the major causes of renal artery thrombosis post-transplantation (1,2). Although proximal renal artery stenosis is previously reported as a cause of renal artery thrombosis post-transplantation, up to our knowledge no case of renal artery thrombosis due to distal renal artery stenosis has been reported before in the literature written in English. In this article, we report a case of renal artery thrombosis that developed early post-transplantation due to distal renal artery stenosis.

## 2. Case presentation

**Figure 1:**
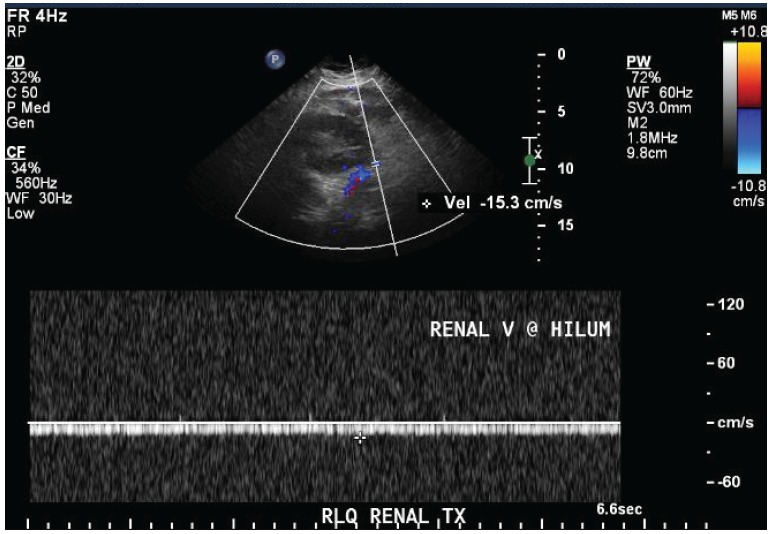
Transplant renal artery Doppler ultrasound showing absent flow.

**Figure 2:**
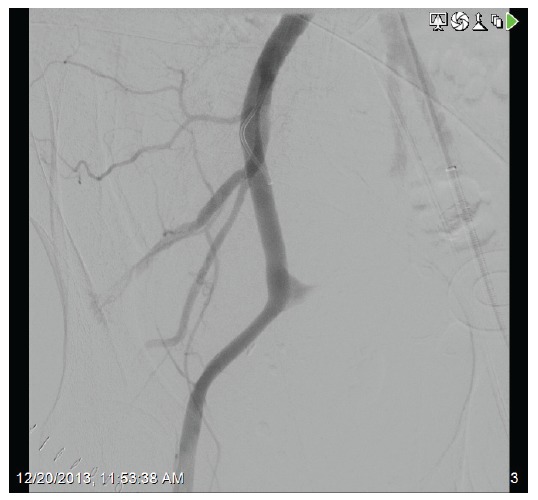
Transplant renal artery angiogram showing renal artery thrombosis.

**Figure 3:**
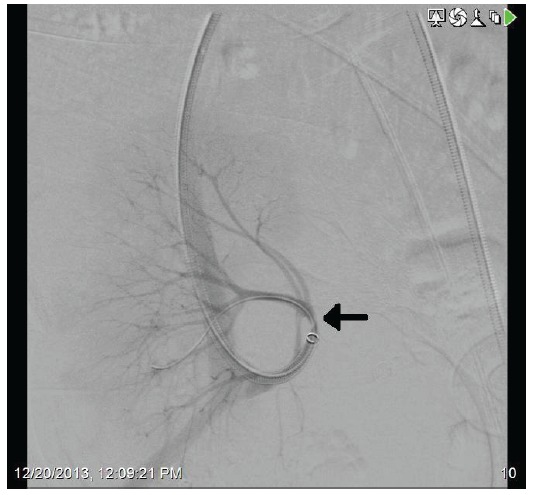
Transplant renal artery angiogram post-thrombectomy showing distal renal artery stenosis (arrow).

**Figure 4:**
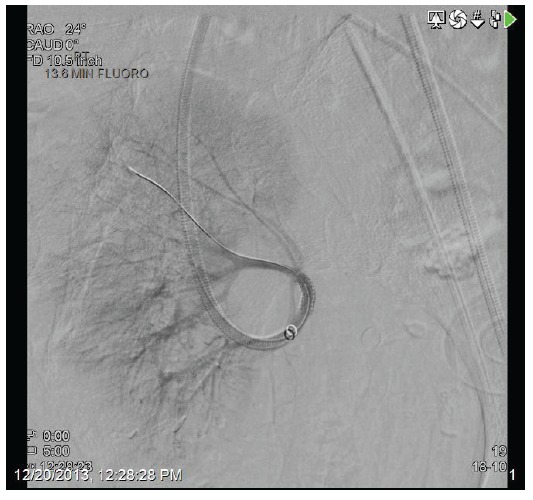
Transplant renal artery angiogram after stenting of distal renal artery stenosis showing patency in entire renal arterial system.

**Figure 5:**
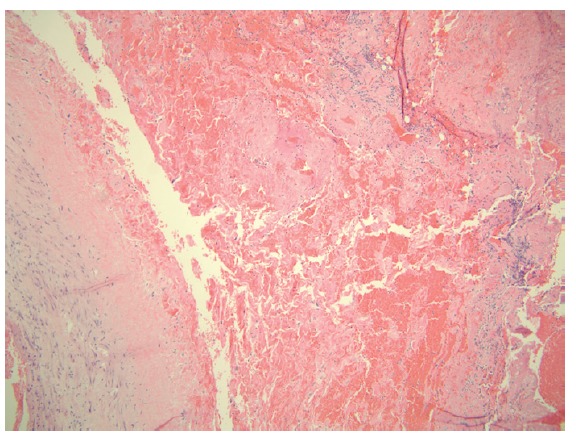
Photomicrograph of the renal artery. The left half of the image displays the muscular wall of the renal artery and the right demonstrates inraluminal organized thrombosis. (Hematoxylin and Eosin stain, original magnification 100X).

**Figure 6:**
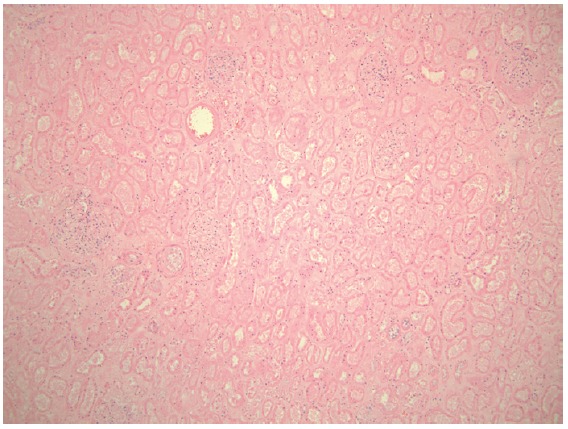
Photomicrograph of renal parenchyma with ischemic necrosis. (Hematoxylin and Eosin stain, original magnification 100X).

A 71-year-old female presented with acute kidney injury 7 days after undergoing a deceased donor kidney transplant. She had end stage kidney disease secondary to diabetes mellitus type 2, and was on peritoneal dialysis prior to transplant. Post-transplant, patient required peritoneal dialysis for first 48 hours due to delayed graft function. The kidney transplant ultrasound with Doppler done 2 days post-transplant showed resistive indices of 0.98 but was otherwise unremarkable. Her serum creatinine had improved to 2.9 mg/dl and 24 hour urine output was up to 1500 ml on the day of discharge (post-operative day 6). Her discharge immunosuppression were tacrolimus 4 mg oral twice daily, mycophenolate sodium 1000 mg oral twice daily, and prednisone 20 mg once daily. On admission, patient complained of nausea, vomiting, reduced urine output, and mild pain at her transplant site. Her serum creatinine had trended up to 3.5 mg/dl. A kidney transplant ultrasound with ecocolordoppler done on admission showed absent arterial and venous blood flow (Figure 1 ). A transplant kidney angiogram was ordered urgently and it showed transplant renal artery thrombosis (Figure 2 ). The occluded artery was catheterized and a thrombectomy was performed. Post-thrombectomy renal angiogram showed distal renal artery stenosis (Figure 3 ) which was dilated and stented successfully. Complete angiogram after stenting showed good blood flow in the entire renal arterial system (Figure 4 ). Patient’s kidney function and urine output did not improve over next 24 hours and repeat renal transplant ultrasound with Doppler showed poor arterial blood flow. Patient underwent transplant nephrectomy and kidney biopsy was obtained. The biopsy showed no evidence of rejection but widespread renal infarction and focal areas of vascular thrombosis (Figure 5  and 6 ). The hypercoagulability work-up was unremarkable and the donor specific antibody was negative.

## 3. Discussion

Renal artery thrombosis in our case most likely occurred due to distal renal artery stenosis. Although the definitive cause of distal renal artery stenosis in our case could not be elicited, it was probably due to injury to the renal artery during back table preparation of the donor kidney. The proximal renal artery stenosis which can develop as a complication of vascular clamp injury, perfusion cannulation injury, or anastomotic dehiscence post-kidney transplantation, is a known cause of renal artery thrombosis (1,2). However, this is the first report of a patient developing renal artery thrombosis secondary to distal renal artery stenosis.

The risk factors for renal artery thrombosis include multiple donor renal arteries, pediatric donors, hypercoagulable states, hypovolemia, prolonged cold ischemia time and delayed graft function (1-4). In addition, peritoneal dialysis pre-transplant has also been reported to be associated with increased incidence of renal artery thrombosis, a risk factor that was also present in our patient.

Renal artery thrombosis usually presents with sudden onset oliguria or anuria accompanied by pain and tenderness over the graft site. Patients may develop thrombocytopenia due to platelet aggregation at the thrombosis site (1). The imaging modality of choice for diagnosis of renal artery thrombosis is color Doppler sonography (1,5). Conventional, computed tomography (CT) and magnetic resonance (MR) angiography may also be used to confirm the presence of renal artery thrombosis. Although there are reports of successful resolution of post-transplant acute renal artery thrombosis with endovascular and surgical modalities such as percutaneous thrombus aspiration, intra-arterial injection of fibrinolytic agents and surgical thrombectomy, renal artery thrombosis usually results in ischemic necrosis and graft loss as was seen in our patient (1,2,6).

## 4. Conclusions

In conclusion, this is the first case report of renal artery thrombosis developing as a complication of distal renal artery stenosis. This important complication should be considered in the differential diagnosis of acute kidney injury occurring immediately post-kidney transplantation.

## Authors’ contributions

All authors contributed equally to the manuscript.

## Conflict of interests

None.

## Funding/Support

None.
